# Isolation, Identification and Screening of Plastic-Degrading Microorganisms: Qualitative and Structural Effects on Poly(Butylene Succinate) (PBS) Films

**DOI:** 10.3390/polym17081128

**Published:** 2025-04-21

**Authors:** Cristina América Morando-Grijalva, Ana Ramos-Díaz, Angel H. Cabrera-Ramirez, Juan Carlos Cuevas-Bernardino, Soledad Cecilia Pech-Cohuo, Angela Francisca Kú-González, Julia Cano-Sosa, Iván Emanuel Herrera-Pool, Sergio Valdivia-Rivera, Teresa Ayora-Talavera, Neith Pacheco

**Affiliations:** 1 Centro de Investigación y Asistencia en Tecnología y Diseño del Estado de Jalisco, A.C. Parque Científico Tecnológico de Yucatán, Km 5.5 Carretera, Sierra Papacal-Chuburna, Chuburna, Merida 97302, Yucatan, Mexico; camorando_al@ciatej.edu.mx (C.A.M.-G.); aramos@ciatej.mx (A.R.-D.); angel_humbert00@hotmail.com (A.H.C.-R.); jcano@ciatej.mx (J.C.-S.); ivherrera_al@ciatej.edu.mx (I.E.H.-P.); tayora@ciatej.mx (T.A.-T.); 2 SECIHTI-Centro de Investigación y Asistencia en Tecnología y Diseño del Estado de Jalisco, A.C. Parque Científico Tecnológico de Yucatán, Km 5.5 Carretera, Sierra Papacal-Chuburna, Chuburna, Merida 97302, Yucatan, Mexico; jcuevas@ciatej.mx (J.C.C.-B.); svaldivia@ciatej.mx (S.V.-R.); 3 Departamento de Ingeniería en Robótica Computacional, Universidad Politécnica de Yucatán, Tablaje Catastral 7193, Carretera, Merida-Tetiz Km 4.5, Merida 97357, Yucatan, Mexico; soledad.pech@upy.edu.mx; 4 Unidad de Biología Integrativa, Centro de Investigación Científica de Yucatan, Merida 97205, Yucatan, Mexico; angela@cicy.mx

**Keywords:** isolation, bacteria, biodegradation, plastics films, poly(butylene succinate) (PBS)

## Abstract

(1) Background: Plastic contamination is on the rise, despite ongoing research focused on alternatives such as bioplastics. However, most bioplastics require specific conditions to biodegrade. A promising alternative involves using microorganisms isolated from landfill soils that have demonstrated the ability to degrade plastic materials. (2) Methods: Soil samples were collected, and bacteria were isolated, characterized, and molecularly identified. Their degradative capacity was evaluated using the zone of clearing method, while their qualitative and structural degradative activity was assessed in a liquid medium on poly(butylene succinate) (PBS) films prepared by the cast method. (3) Results: Three strains—*Bacillus cereus* CHU4R, *Acinetobacter baumannii* YUCAN, and *Pseudomonas otitidis* YUC44—were selected. These strains exhibited the ability to cause severe damage to the microscopic surface of the films, attack the ester bonds within the PBS structure, and degrade lower-weight PBS molecules during the process. (4) Conclusions: this study represents the first report of strains isolated in Yucatán with plastic degradation activity. The microorganisms demonstrated the capacity to degrade PBS films by causing surface and structural damage at the molecular level. These findings suggest that the strains could be applied as an alternative in plastic biodegradation.

## 1. Introduction

Plastics are synthetic polymeric materials that become flexible when heated and can therefore be molded into various objects. They are composed of elements such as carbon, hydrogen, silicon, oxygen, chlorine, and nitrogen [1]. The most widely used plastics are known as commodities, a type of thermoplastic produced in large quantities at low cost. This group includes polyethylene (PE), polypropylene (PP), polyvinyl chloride (PVC), polystyrene (PS), and polyethylene terephthalate (PET) [2]. However, the extensive use of primarily single-use plastic products and inadequate plastic waste management by the plastics industry [3] have led to their accumulation in the environment. These materials persist for long periods because they are difficult to degrade; for example, PET, PVC, and PS can have a half-life exceeding 2500 y, while high-density polyethylene can have a half-life from 250 to 5000 years [4]. Their presence in the environment poses significant risks, potentially causing irreparable harm to wildlife [5,6,7] and impacting human health [8].

Consequently, recent research and industry efforts have focused on exploring alternatives and to replace synthetic plastics with bioplastics, which offer significant advantages, such as a drastic reduction in environmental pollution [9,10,11]. These materials, being biodegradable, align with the principles of the circular economy, which promotes the sustainable production and disposal of materials. Biodegradation (under specific conditions) offers greater advantages than physical and chemical recycling. Although recycling processes have reported good yields in monomer recovery, they often require high temperatures, solvents, and the release of components that may necessitate additional treatment steps due to their toxicity [12]. However, it has been noted that bioplastics are not always biodegradable, or they may require specific conditions, such as industrial composting, for effective decomposition [13]. Additionally, if improperly managed and sent to landfills, bioplastics can still be pollutants, releasing environmentally harmful substances during degradation [14,15,16,17].

Poly (butylene succinate) (PBS) is an aliphatic polyester composed of succinic acid and 1,4-butanediol monomers. It is traditionally synthesized from fossil-based raw materials but has recently been produced from renewable sources such as biomass [18]. One of the primary applications of this polymer is in the food packaging industry, where its mechanical and barrier properties have been enhanced through the incorporation of other components and polymers [19,20]. However, while PBS is biodegradable in various environments, it may not degrade effectively under anaerobic conditions [21], which limits its effectiveness as a sustainable alternative.

Given this challenge, microorganisms capable of biodegrading plastics have emerged as a promising alternative and have been isolated from various environments. Recent studies have reported that bacteria from soil and landfill sites, belonging to genera such as *Bacillus* [22,23,24,25], *Pseudomonas* [13,15], and *Acinetobacter* [26], among others, possess the capacity to degrade plastics, including PE, PET, PS, PP, and bioplastics such as polycaprolactone (PCL). However, the degradation percentages remain low, often requiring prolonged exposure times (up to 1000 days), elevated temperatures (40–60 °C), or the application of physical or chemical pretreatments to achieve significant or complete biodegradation [14,15,23,24,27]. In the case of PBS, which is classified as a hydrolytic polyester, its biodegradation is facilitated by microorganisms that produce enzymes such as esterases, which cleave the ester bonds in its structure [28]. This process breaks PBS down into simpler compounds like carbon dioxide and water, leading to observable changes in surface properties, mechanical properties, and overall performance [28]. Šašinková [29] demonstrated that PBS can be mineralized by up to 80% over a period of 180 days at 37 °C using activated sludge from wastewater treatment plants as an inoculum in simulated freshwater environments. However, in the same study, one replicate exhibited a much slower degradation rate, achieving only 2% mineralization [29]. Consequently, the process remains inefficient and economically unviable at present.

Finally, the techniques used to evaluate this process are crucial for determining the effectiveness of biodegradation and assessing the required conditions. Various methods, such as Fourier transform infrared spectroscopy (FTIR), gas chromatography, thermogravimetric analysis, and scanning electron microscopy (SEM), have been employed to monitor changes in both the chemical structure and surface properties of polymers [30]. However, many of these techniques are destructive, require additional sample preparation, are costly, or have low sensitivity to subtle structural changes [30]. Conversely, Atmospheric Solids Analysis Probe (ASAP)–mass spectrometry has been utilized to comprehensively study the composition and structure of polymeric materials, providing significant results that validate plastic degradation [30]. When used together, these technologies could play a key role in evaluating plastic degradation and elucidating degradation mechanisms [31]. Therefore, the main objectives of this study were (a) to isolate, identify, and screen plastic-degrading microorganisms from microbial consortia found in plastic waste in Yucatán and (b) to evaluate the degradative capacity of the isolated strains on polybutylene succinate (PBS) films using SEM, FTIR, and ASAP–mass spectrometry as the primary analytical methods.

## 2. Materials and Methods

### 2.1. Collection and Isolation of Microbial Consortia

Microorganisms were isolated from soil samples collected in the state of Yucatán, Mexico. The inclusion criteria focused on soils with visible plastic residue accumulation and signs of initial degradation. The samples were collected from the following areas: Sierra Papacal (21°11′29.5″ N, 89°49′11.3″ W), Chuburná Puerto (21°13′25.6″ N, 89°49′45.8″ W), and Yucalpetén (21°16′36.8″ N, 89°42′39.8″ W) in Yucatán (Figure S1). Soil samples were obtained using a soil sampler at a depth of approximately 5–10 cm [32]. About 200 g of each sample was collected and placed in autoclaved plastic bags. The sampler was cleaned with 70% ethanol after each sampling. Samples were then transported to the laboratory for further analysis [33].

The methodology for microorganism isolation followed the protocol reported by Rakesh [34], with some modifications. A total of 5 g of soil was resuspended in 10 mL of sterile peptonized water, and serial dilutions were prepared up to 10^−4^ (g/mL). From the final dilution, 100 µL was inoculated by spreading on Petri dishes containing Actinomycete Isolation Agar (AIA) culture medium (HIMEDIA, Kennett Square, PA, USA) and incubated at 28 °C until growth was observed. Pure colonies were then inoculated onto nutrient agar (NA) plates (composed of casein peptone 5.0 g/L, heart–brain infusion 1.0 g/L, yeast extract 2.0 g/L, NaCl 5.0 g/L, and bacteriological agar 15.0 g/L), supplemented with 0.1 µg/mL nystatin and 5 µg/mL carbenicillin. The Petri dishes were incubated at 28 °C for 24 h in a bioclimatic chamber. Purified colonies were maintained on ISP2 medium (composed of yeast extract 4.0 g/L, malt extract 10.0 g/L, dextrose 4.0 g/L, and bacteriological agar 20.0 g/L) [35].

### 2.2. Characterization of Isolated Microorganisms

The cell morphology of the isolated strains was evaluated using phase-contrast microscopy after staining with methylene blue [36]. Biochemical characterization included Gram staining [37] and a catalase test, which was conducted using the slide (droplet) method described by [38]. Additionally, spore production was assessed using malachite green staining [39].

### 2.3. Molecular Identification of Isolated Microorganisms

Genomic DNA from the isolated microorganisms was extracted using the PureLink^®^ Genomic DNA Kit (K1820-02, Invitrogen, Thermo Fisher Scientific Inc, Waltham, MA, USA), following the manufacturer’s instructions. The purity of the genomic DNA was verified by 1% agarose gel electrophoresis, performed at 70 V for 1 h using 1X TAE buffer. Gels were stained with ethidium bromide and visualized under UV light using the UVP Gel Doc Ez system (Gel Doc Ez, Bio-Rad Laboratories, Inc, Hercules, CA, USA).

Universal 16S rDNA primers (27F: 5′-AGAGAGTTTGATCCTGGCTCAG-3′ and 1492R: 5′-GGTTACCTTGTTACGACT-3′) were used to amplify the 16S rRNA gene fragment. The PCR conditions consisted of an initial denaturation at 96 °C for 2 min, followed by 30 cycles of denaturation at 95 °C for 30 s, annealing at 55 °C for 30 s, and extension at 72 °C for 30 s. A final extension step was performed at 72 °C for 10 min. The amplified PCR products were verified by 1% agarose gel electrophoresis, stained with ethidium bromide, and visualized using the UVP Gel Doc Ez system (Gel Doc Ez, Bio-Rad Laboratories, Inc, Hercules, CA, USA).

The PCR products were purified using the PureLink^®^ PCR Purification Kit, Invitrogen (Thermos Fisher Scientific Inc., Waltham, MA, USA) following the manufacturer’s instructions, and sent to Macrogen Inc. for Sanger sequencing in both directions. The obtained sequences were analyzed using the NCBI BLAST tool. The sequences were aligned with reference sequences using the CLUSTAL OMEGA program [40]. A phylogenetic tree was constructed using the MEGA software (version 11.0) based on the neighbor-joining method, with statistical support provided by a bootstrap analysis of 1000 replicates.

### 2.4. Selection of Degrading Microorganisms by the Zone of Clearing Method

The strain was precultured in test tubes containing 5 mL of the basal medium (PBM) (patent application MX/a/2023/015172 [41]), consisting of yeast extract (0.25 g/L), magnesium sulfate heptahydrate (0.2 g/L), sodium chloride (0.1 g/L), calcium chloride dihydrate (0.02 g/L), ferrous sulfate heptahydrate (0.01 g/L), manganese sulfate (0.0005 g/L), and agar (15.0 g/L). The cultures were incubated at 37 °C with constant shaking for at least 24 h. Petri dishes with two compartments were prepared by adding MBP medium containing emulsified commercial PBS (0.05%). The dishes were inoculated using sterile filter paper discs impregnated with the precultured strain and placed in the center of one compartment. For the blank control, sterile filter paper discs impregnated with sterile basal medium were used and placed in the center of one compartment. An *Escherichia coli* strain was used as a negative control [42]. The Petri dishes were incubated at 37 °C throughout the study, and all treatments were conducted in triplicate.

The degradative activity was assessed by observing the formation of a halo or clear zone around the paper discs. The diameter of the halo was measured in millimeters every 3 to 6 h for 10 days to determine the extent of degradation.

### 2.5. PBS Film Preparation

The films were prepared using the casting method based on the methodology established in the patent application MX/a/2023/015172 [41]. A 5% (w/v) forming solution was prepared in acetone, maintained at 50 °C, and stirred continuously until a homogeneous solution was obtained. The solution was then placed in a sonic bath for 10 min. Afterward, it was poured into beakers at a density of 0.122 g/cm^2^ and left to evaporate for 24 h in a fume hood with the airflow turned off. The resulting films measured 2 × 0.5 cm, displayed a white color, and had a heterogeneous appearance, with a thickness of 0.057 ± 0.011 mm [43].

#### Film Conditioning

The films were conditioned according to the guidelines established by the American Society for Testing and Materials (ASTM) [44]. They were placed in a desiccator and maintained at a temperature of 25 ± 2 °C with a relative humidity of 50% (using sodium bromide beads) for 40 h.

### 2.6. Biodegradation of PBS Films in Liquid Medium

The films were immersed in 70% alcohol followed by sterile distilled water. They were then placed in 15 mL Falcon tubes containing 5 mL of MBP. Bacteria in the exponential phase were added to achieve an initial optical density (OD) of 0.2. The samples were kept under agitation at 37 °C for 21 weeks, with samples collected every 7 weeks (patent application MX/a/2023/015172 [41]).

#### Plastic Film Recovery

At the conclusion of the experiments, the films were recovered by filtration using filter paper. The films were washed with a 2% sodium dodecyl sulfate (SDS) solution, followed by washing with warm sterile distilled water (40 °C) for 20 min. They were then left to dry overnight at room temperature (near 27.4 °C) and subsequently placed in a drying oven (model 9162, ECOSHEL, Pharr, TX, USA) at 35 °C for 48 h. Afterward, the corresponding analyses were performed.

### 2.7. Morphological and Structural Analysis of Films and Their Degradation

#### 2.7.1. Macroscopic and Microscopic Characterization of Plastic Films

To evaluate macro morphology, photographs of the films were taken using HUAWEI P30 Lite mobile phone (sensor 24 MP 1:1.8/27; HUAWE, Shenzhen, China) on a dark background to contrast any observable macroscopic changes. Meanwhile, microscopic surface examination of the films was performed using scanning electron microscopy (SEM) following the methodology reported by Tribedi [45] with some modifications. Briefly, film samples approximately 0.5 cm × 0.5 cm in size were taken (some of the recovered films were fragmented) and fixed in 4% formaldehyde. The samples were then subjected to a dehydration process involving sequential immersion in ethanol solutions at concentrations of 30%, 50%, 70%, 80%, 90%, 96%, 100%, 100%, and 100%, with each step lasting one hour. Solution changes were carried out by decantation, ensuring the samples were not lost. The samples were stored under refrigeration until analysis.

The films were mounted on metallic sample holders using double-sided carbon tape and silver paint. They were then coated with a thin layer of gold and observed under a JEOL scanning electron microscope (model JSM-6360 LV, JEOL, Akishima, Japan) operating under low vacuum conditions and using an electron beam set to 20 kV (100, 20, 10, and 1 µm at 150×, 900×, 1500×, and 12,000× magnification used for observation, respectively).

#### 2.7.2. Fourier Transform Infrared Spectroscopy (FT-IR)

Infrared spectra were obtained using a Thermo Scientific Nicolet iS5 infrared spectrometer (Thermo Fisher Scientific Inc., Waltham, MA, USA) equipped with an Attenuated Total Reflection (ATR) accessory (with a diamond-ZnSe crystal). The samples were scanned across the spectral range of 4000 to 600 cm^−1^. The sample preparation process is described in Section Plastic Film Recovery.

#### 2.7.3. Structural Analysis Using Atmospheric Solids Analysis Probe–Mass Spectrometry (ASAP-MS)

A Waters Xevo-TQs micro mass spectrometer (Waters Corporation, Milford, MA, USA) equipped with a Waters Atmospheric Solids Analysis Probe (Part number 715003410, Waters Atmospheric Solids Analysis Probe, Milford, MA, USA) was used for the analysis. The analysis was conducted in negative polarity under the following conditions: cone voltage (50 V), corona current (23.10 µA, 2.70 kV), source temperature (120 °C), desolvation temperature (100–650 °C), desolvation gas flow (nitrogen, 500 L/h), and cone gas flow (0 L/h). Additionally, cone voltages between 25 and 150 V were applied, with a collision energy of 0 eV. Mass spectra were recorded in full scan mode, covering a range of 50 to 2048 m/z. A desolvation temperature gradient was employed according to the following program: 0–1 min: 100 °C, 1–2 min: 150 °C, 2–3 min: 200 °C, 3–4 min: 300 °C, 4–6 min: 400 °C, 6–8 min: 500 °C, and 8–15 min: 625 °C. For the PBS degradation analysis, Capillary Tubes Soda Glass (G119/32 [length: 100 mm; outlet diameter: 1.8 mm]; S. Murray & Co., England, UK), pre-treated at 600 °C for 1 min to eliminate any potential interference, were used as the sample contact surface and introduced directly into the ASAP system. Data acquisition and processing were performed within the range of 8–9.5 min using MassLynx V4.1 software (Waters, Milford, MA, USA).

### 2.8. Statistical Analysis

Data were expressed as the mean ± standard deviation of at least three independent experiments. Using the statistical software Statgraphics Centurion XVI.I (Statgraphics Technologies Inc., The Plains, VA, USA), a one-way analysis of variance (ANOVA) was performed followed by a mean comparison test with a significance level of *p* ≤ 0.05.

## 3. Results and Discussion

From the collected soil, 14 strains were isolated and purified in culture media. The biochemical characterization was performed for each strain, and the results are presented in Table S1. The obtained characteristics were compared with the molecular identification.

### 3.1. Molecular Identification of the Isolated Strains

After DNA extraction from the 14 isolated strains, the amplified fragments were sent to Macrogen Inc. (Seoul, Republic of Korea) for sequencing. The sequences obtained were of high quality for all 14 strains, enabling further screening using the GenBank database [46] for sequence similarities. Significant matches (>99% similarity) were identified for the isolated strains, as presented in Table 1.

The isolated bacteria were classified into the genera *Bacillus*, *Enterobacter*, *Acinetobacter*, *Klebsiella*, and *Pseudomonas*, as shown in Table 1. Among the identified strains, five displayed high-quality consensus sequences with identity confirmed at the species level. These strains were registered with the National Center for Biotechnology Information (NCBI) with accession numbers *Bacillus cereus* CHU4R, accession number ON721265; *Enterobacter hormaechei* MORI66, accession number ON721279; *Acinetobacter baumannii* YUCAN, accession number ON721281; *Klebsiella pneumoniae* MORI33, accession number ON721265; *Pseudomonas otitidis*, accession number ON721280.

**Figure 1:**
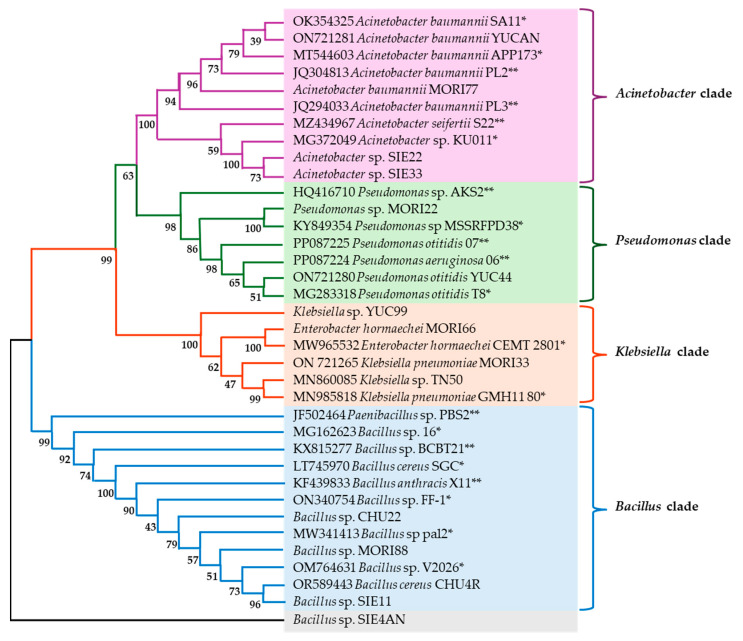
Bacterial relationship cladogram of presumptively identified strains, including reference strains for identity (*) and strains with documented plastic-degrading capacity (**). The purple, green, orange and blue sections highlight the clades formed by each genus.

Molecular identification enabled the construction of a cladogram (Figure 1) that relates the sequences of the isolated strains to reference strains of known identities and those with demonstrated degradative capacity for various plastics, including biodegradable aliphatic polymers. In that sense, the cladogram was constructed based on sequence similarity found through the Basic Local Alignment Search Tool (Basic Local Alignment Search Tool, BLAST (version 2024)), incorporating sequences from bacterial strains previously reported to have plastic-degrading capabilities (Figure 1).

The cladogram analysis revealed the formation of four main clades corresponding to genera: *Acinetobacter*, *Klebsiella*, *Pseudomonas*, and *Bacillus*, which formed several clusters. For example, the first clade includes the strains *A. baumannii* MORI77 and *A. baumannii* YUCAN grouped within the same clade with the reference strains OK354325 *A. baumannii* SA11, MT544603 *A. baumannii* APP173, JQ304813 *A. baumannii* PL2, and JQ294033 *A. baumannii* PL3 [47], the latter of which was isolated from municipal dumpsite soil and demonstrated degradative activity against low-density polyethylene (LDPE) films. Similarly, the strains *Acinetobacter* sp. SIE22 and *Acinetobacter* sp. SIE33 clustered with MZ434967 *A. seifertii* S22, a strain isolated from soil by [42], which exhibited degradative capacity for polycaprolactone (PCL) films, and in turn with the reference strain MG372049 *Acinetobacter* sp. KU 011.

The second clade comprises the strains *Pseudomonas* sp. MORI22 grouped within the same clade with the reference strains KY849354 *Pseudomonas* sp. MSSRFPD38 and ON721280 *P. otitidis* YUC44, which are closely related to PP087224 *P. aeruginosa* O6 and PP087225 *P. otitidis* O7, both of which were isolated from sediment by [48] and evaluated for their ability to degrade micro- and nanoplastics of PE, PS, and PET and grouped within the same clade with the reference strain MG283318 *P. otitidis* T8. Additionally, the strain HQ416710 *Pseudomonas* sp. AKS2, isolated from soil and capable of degrading polyethylene succinate (PES) films, appears as an independent branch within this clade [45].

The third clade, the strains *Klebsiella* sp. YUC99 and ON721265 *K. pneumoniae* MORI33 are grouped together, with the latter clustering alongside the reference strains MN860085 *Klebsiella* sp. TN50 and MN985818 *K. pneumoniae* HMH1180. This clade also includes ON721279 *Enterobacter hormaechei* MORI66, which is strongly associated with MW965532 *E. hormaechei* CEMTC 2801; both genera belong to the *Enterobacteriaceae* family.

The fourth clade includes the strains *Bacillus* sp. CHU22, *Bacillus* sp. MORI88, *Bacillus* sp. SIE11, and OM764631 *B. cereus* CHU4R, which are grouped with the strain JF502464 *Paenibacillus* sp. PBS-2, isolated from marine sand and capable of degrading PBS [49]. These strains are also strongly related to KX815277 *Bacillus* sp. BCBT21, which was isolated from the composting process of agricultural plastic waste and has been shown to degrade PP plastic bags [50], and to KF439833 *B. anthracis* X11, a strain isolated from landfill soil by [51] that has demonstrated the ability to degrade LDPE. Meanwhile, *Bacillus* sp. SIE4AN forms an independent but closely related branch within the *Bacillus* clade.

As mentioned above, the cladogram indicated that most of the isolated strains were closely related to microorganisms known for their capacity to degrade various types of plastics and bioplastics, including those used for bag or packaging material production [50]. However, few studies have reported PBS degradation at the species level. Therefore, this study could be the first to document this degradative activity at such a level.

Based on these findings, the strains *A. baumannii* YUCAN, *P. otitidis* YUC44, and *B. cereus* CHU4R were selected for further testing. These strains were registered and deposited at the National Center for Genetic Resources (CNRG), in Mexico), with the following accession numbers: *A. baumannii* YUCAN CM-CNRG TB246, *P. otitidis* YUC44 CM-CNRG TB245, and *B. cereus* CHU4R CM-CNRG TB244.

### 3.2. Evaluation of the Degradation Activity of Selected Strains Using the Clear Zone Method

**Figure 2:**
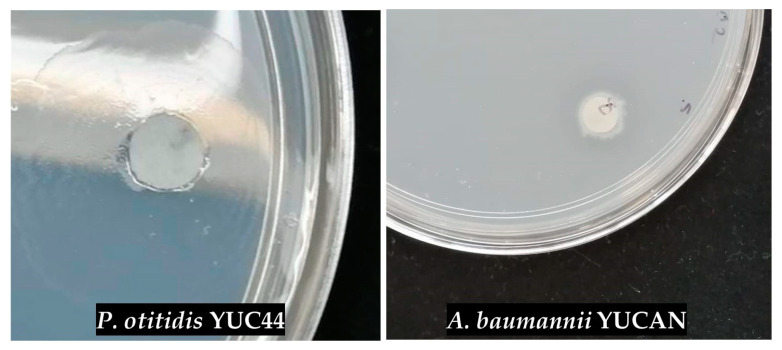
Formation of clear zones by *P. otitidis* YUC44 and *A. baumannii* YUCAN on Petri dishes containing PCL emulsified in MBP.

After the molecular identification of the strains and their relationship with reference strains capable of degrading plastics, their degradative activity was evaluated using the clear zone method (Figure 2). This method allows for the visualization and assessment of each strain’s degradation capacity, establishing a direct correlation between the selected genetic profiles and their efficiency in polymer biodegradation.

The initial degradation test was conducted with the strains *Acinetobacter* sp. SIE33, *Acinetobacter* sp. SIE22, *Klebsiella* sp. YUC99, *Bacillus* sp. SIE4AN, *Pseudomonas otitidis* YUC44, and *Acinetobacter baumannii* YUCAN on Petri dishes containing 15% PCL emulsified in basal medium. The formation of a clear zone was monitored. Although colony growth was observed for all strains, only *P. otitidis* YUC44 and *A. baumannii* YUCAN formed clear zones after 6 and 14 days of incubation, respectively, at a temperature of approximately 25.5 °C (Figure 2).

**Figure 3:**
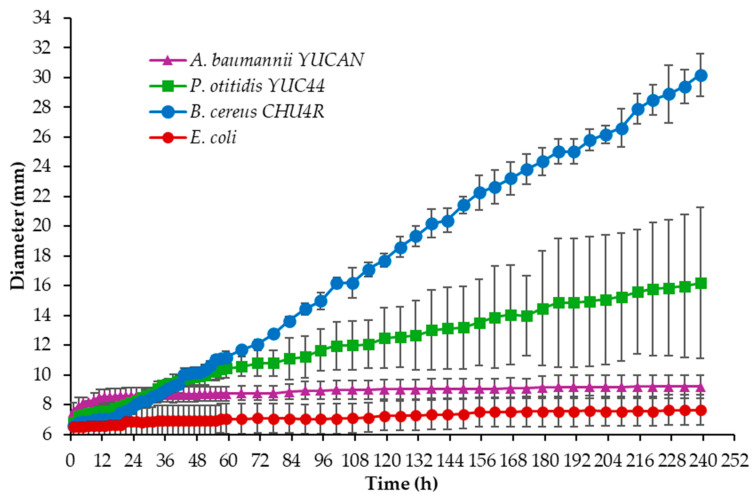
Changes in clear zone/colony diameter of the isolated strains over 238 h at room temperature on Petri dishes containing PBS emulsified in MBP.

Additionally, the degradation capacity was tested on Petri dishes containing commercial PBS emulsified in MBP, with *Escherichia coli* serving as a negative control [42,45], with no reports in the literature indicating that *E. coli* can degrade plastics. Measurements of growth and halo formation were recorded every 2 h for the first 59 h and, subsequently, every 6 h. As shown in Figure 3, the strains *A. baumannii* YUCAN, *P. otitidis* YUC44, and *B. cereus* CHU4R reached diameters of 9.21 ± 0.79 mm, 16.19 ± 5.11 mm, and 30.19 ± 1.45 mm, respectively, while *E. coli* showed a diameter of 7.64 ± 0.52 mm after 239 h of incubation. The result for *B. cereus* CHU4R was slightly higher than that reported by [52], who isolated bacteria from the seafloor and evaluated their degradative capacity on emulsified PBS in a marine medium supplemented with glucose as a carbon source (incubating at 30 °C) and observed clear zones of approximately 12 mm in radius after 7 days of culture with *Terribacillus* sp. JY49. In contrast, the present study used PBS films as the sole carbon source and maintained incubation at room temperature (approximately 35–40 °C).

The results obtained from the strains *A. baumannii* YUCAN, *P. otitidis* YUC44, and *B. cereus* CHU4R surpass those reported by Suzuki [53], who isolated the *Nocardioides* OK12 strain from marine plastic waste and evaluated its degradation capacity on various bioplastics, including polycaprolactone (PCL) and poly(3-hydroxybutyrate) (PHB). They observed the formation of halos larger than 8 mm in Petri dishes with PHB at 30 °C, while no growth or halo formation was detected on the PCL plates.

It has been reported that *Pseudomonas* sp. AKS2 can grow in a medium emulsified with PES, forming a clear zone around the colony after 10 days of incubation at 30 °C [45]. More recently, *P. mendocina* SA-1503, isolated from landfill soil, demonstrated the ability to degrade emulsified polyesters such as PBS, P34HB, and PCL, producing a larger clear halo in the presence of PBS [27]. Similarly, [42] isolated *A. seifertii* S22 from landfill soil using Petri dishes with emulsified PCL, observing a 25.0 ± 1.0 mm clear halo and a 7.1 ± 0.0 mm colony growth at 37 °C over five days.

In the present study, it was not possible to calculate the degradation index because the colonies grew at a similar rate to the clear zone. However, the results obtained with the *B. cereus* CHU4R strain exceeded those reported for *Bacillus* sp. JY35, which was isolated from sediment and evaluated on poly(butylene adipate-co-terephthalate) (PBAT) films. In that study, a clear zone diameter of less than 25 mm was observed after 10 days of incubation at 30 °C [54].

The results obtained through the clear zone method provide a preliminary assessment of the isolated strains’ ability to degrade plastics, consistent with findings reported in the literature. However, the degradation experiment in a liquid medium enabled a more precise evaluation of the strains’ degradative activity by analyzing the structural changes in the treated plastic films.

### 3.3. Liquid Biodegraded Film Characterization

#### 3.3.1. Macroscopic and Microscopic Characterization by Scanning Electron Microscopy (SEM)

**Figure 4:**
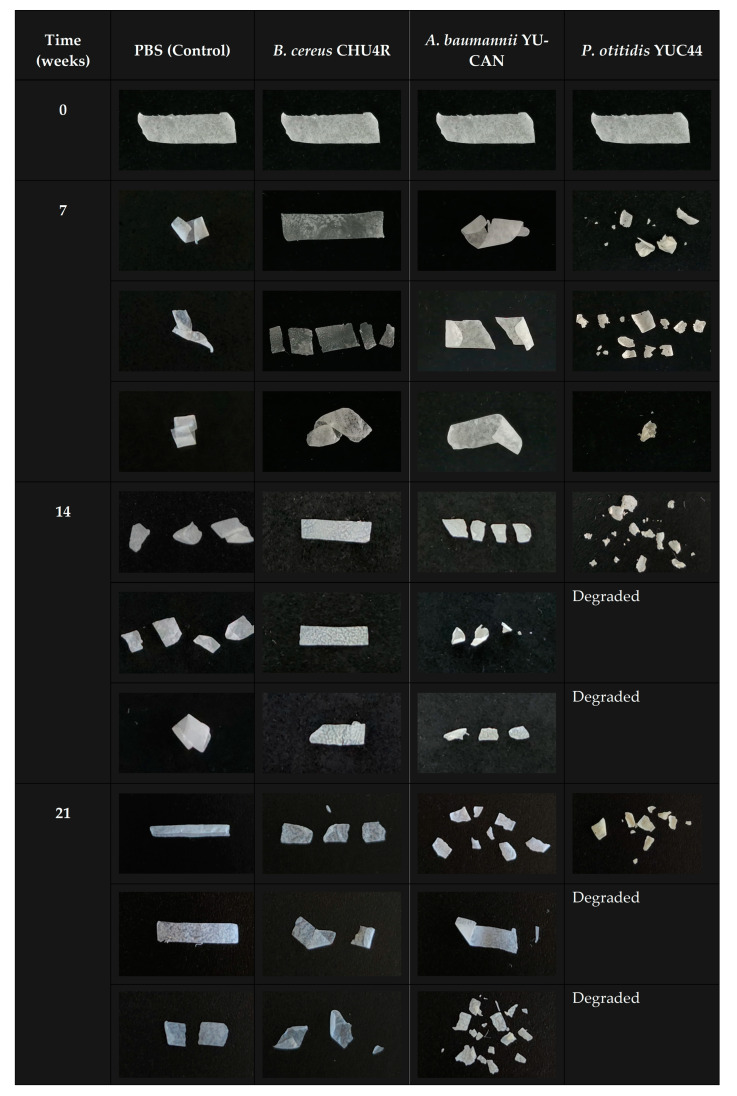
Changes in PBS films during biodegradation in liquid MBP over 21 weeks at 37 °C.

Figure 4 shows the changes in PBS films during biodegradation in liquid MBP over 21 weeks at 37 °C. Overall, it was observed that some films became brittle and adopted a “curled” or “caked” shape over time. In certain images, it appears as though only part of the film is shown, but this is due to the films curling upon themselves (notably, in the control and *B. cereus* CHU4R samples) (Figure 4). After 7 weeks of incubation, *B. cereus* CHU4R and *A. baumannii* YUCAN showed only fragmented film remnants, while *P. otitidis* YUC44 presented partial films with approximately 45% degradation. By week 14, films exposed to *B. cereus* CHU4R displayed substantial damage, with *A. baumannii* YUCAN showing completely fragmented films. In contrast, *P. otitidis* YUC44 presented fragments from only one film, while no remaining fragments were recovered from the others, possibly indicating complete degradation. At the 21-week mark, further damage was observed, with an increased number of film particles in the *B. cereus* CHU4R and *A. baumannii* YUCAN treatments. The condition of the *P. otitidis* YUC44-treated films remained similar to that at 14 weeks.

**Figure 5:**
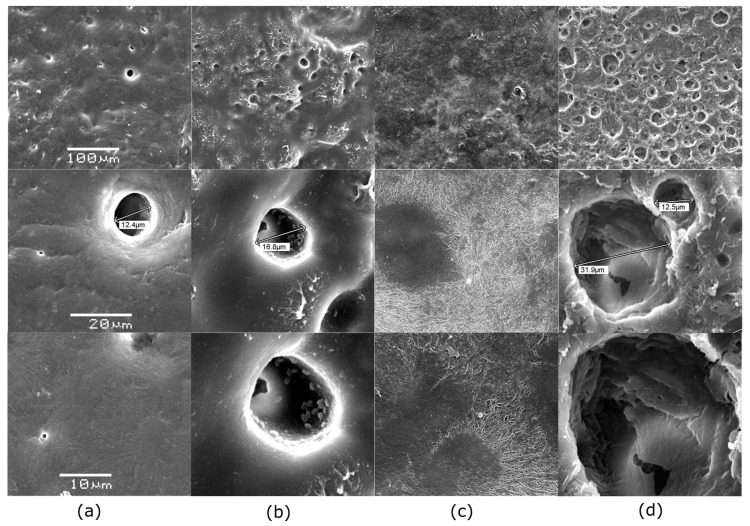
Micrographs of film surfaces observed under SEM after 21 weeks of treatment with (**a**) control PBS, (**b**) *B. cereus* CHU4R, (**c**) *A. baumannii* YUCAN, and (**d**) *P. otitidis* YUC44.

**Figure 6:**
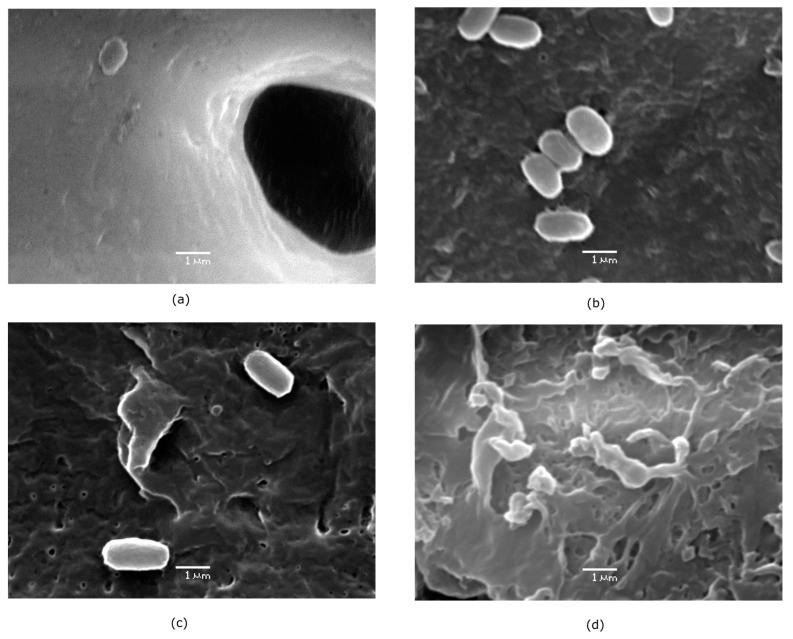
Commercial PBS (**a**) and evidence of biofilm formation at different stages on the surface of PBS films, showing the morphology of adherent cells from (**b**) *B. cereus* CHU4R, (**c**) *A. baumannii* YUCAN, and (**d**) *P. otitidis* YUC44.

At the microscopic level, Figure 5 presents SEM micrographs of the PBS film used as the control at a 100 µm scale, revealing a surface with reliefs and an irregular texture. When magnified further, pores approximately 12 µm in diameter are visible. At a 10 µm scale (Figure 5), the control film exhibits a uniform texture without signs of erosion. Films treated with *B. cereus* CHU4R (Figure 5) display an increase in pore size (from approximately 12.4 to 16.8 µm) and the formation of new holes where adherent cells are visible, possibly due to the presence of pili (Figure 6). In contrast, films treated with *A. baumannii* YUCAN show a slightly eroded surface with expanding cracks forming a distinctive pattern (Figure 5). Colonization by cells adhered to the surface is also evident, suggesting the early stages of the degradation process [55]. Films exposed to *P. otitidis* YUC44 show extensive damage, with larger and deeper holes (ranging from approximately 12.5 to 31.9 µm) and significant surface cracks (Figure 5), indicating advanced biodegradation. Additionally, biofilm formation (the second stage of degradation) is observed, characterized by the development of microcolonies and possible exopolysaccharide production. These exopolysaccharides coat the cells, serving as a method of anchorage and cell attachment (Figure 6) [56].

This is similar to what was reported by Dang [50], who evaluated the degradation capacity of *Bacillus* sp. BCBT21 on biodegradable plastic bags in a liquid medium at 55 °C for 30 days, observing the formation of holes and layers on the surface. In our study, the results were obtained at room temperature (37 °C), which implies a reduction in the cost of the waste treatment process. In another study, [54] tested a strain of *Bacillus* sp. JY35 and observed the gradual degradation of PBAT plastic film, with the appearance of cracks after 5 days and significant surface damage after 10 days. Conversely, in the present study, PCL films treated with *A. seifertii* S22 exhibited a rough surface with holes and cracks extending across the film. The results obtained with *P. otitidis* YUC44 were similar to those reported by Gupta [15], who evaluated the degradative capacity of *P. aeruginosa* ISJ14 on low-density PE films. After 60 days, they observed the formation of cracks and erosion on the film surface as well as the development of a biofilm that facilitated polymer degradation.

Furthermore, [27] evaluated the biodegradation of PBS films using purified extracellular esterases from *P. mendocina* SA-1503, observing the appearance of cracks and holes on the film surface at 40 °C for 60 h that increased and deepened over time. This suggests that the enzymes produced by *P. otitidis* YUC44 are primarily esterases, as supported by the ATR-FTIR structural analysis, which showed a decrease in the intensity of the bond assigned to the ester functional group. Additionally, the presence of cells adhering to and bonding with one another on the surfaces of films treated with *B. cereus* CHU4R and *A. baumannii* YUCAN was observed, possibly due to pili involved in biofilm formation. In the case of *P. otitidis*, microcolonies were observed, indicating a mature biofilm, similar to findings reported by Tribedi [45], who demonstrated that *Pseudomonas* sp. AKS2 can adhere to polyethylene succinate (PES) films and efficiently degrade the polymer.

#### 3.3.2. FTIR

**Figure 7:**
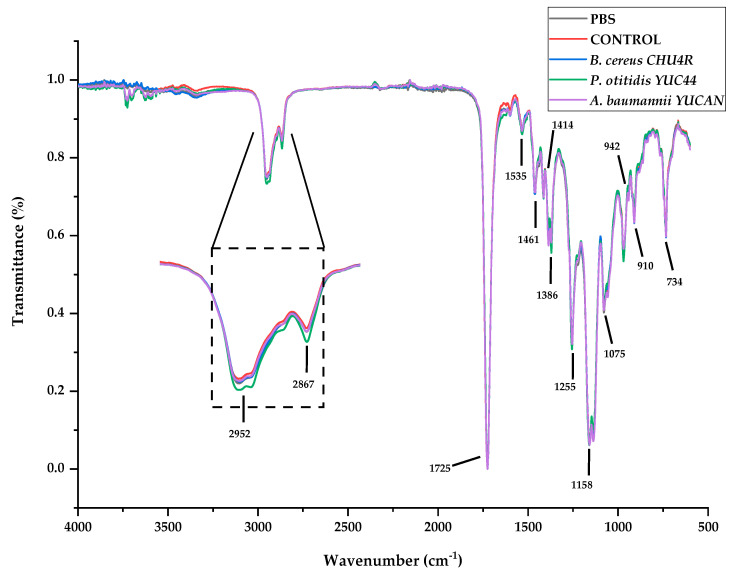
Infrared spectra of PBS films after 21 weeks of treatment. The region highlighted with dotted lines delineates the areas considered in the analysis.

Regarding the vibrational characterization of the films, Figure 7 shows the characteristic bands of commercial PBS plastic present in both the control samples and the films treated with microorganisms after 21 weeks of treatment. Absorption bands were observed at 2952 and 2867 cm^−1^, attributed to the asymmetric and symmetric vibrations of the C-H_2_ and CH_3_ bonds. An intense band was detected at 1725 cm^−1^, corresponding to the stretching of the C = O bond in the carbonyl group, which is characteristic of ester formation and the amorphous region of the polymer [20]. Subtle bands were observed at 1461 and 1414 cm^−1^, corresponding to the deformation and stretching vibrations of the -CH- groups [57]. The asymmetric bending of CH was associated with the band at 1386 cm^−1^ [58], while the intense band at 1158 cm^−1^ was attributed to C-O-C stretching in the repeating -OCH_2_CH_2_- unit [59]. The band at 1075 cm^−1^ corresponded to the symmetric stretching of the ester group [58], and finally, a band at 942 cm^−1^ was identified as -C-OH bending from terminal acid groups [20].

In the spectra regions corresponding to the bands at 2952 and 2867 cm^−1^, a slight shift in the band associated with the treatment involving *P. otitidis* YUC44 was observed (Figure 7). The sum of the peak areas detected in this region was significantly lower (*p* < 0.05) for *P. otitidis* YUC44 (115.44 ± 0.00) and *B. cereus* CHU4R (115.50 ± 0.54) compared with the control (116.90 ± 0.23) and *A. baumannii* YUCAN (117.29 ± 0.32). Changes in peak intensity at 2952 cm^−1^ indicate variations in alkane groups [52].

Additionally, a statistically significant (*p* < 0.05) decrease in the absorbance intensity of the carbonyl group peak at 1725 cm^−1^ was observed in the films treated with *P. otitidis* YUC44, *A. baumannii* YUCAN, and *B. cereus* CHU4R, with values of 0.866 ± 0.00, 0.871 ± 0.02, and 0.877 ± 0.00, respectively, whereas the control exhibited an absorbance of 0.877 ± 0.00.

These results suggest that the microorganisms exhibit different degradation mechanisms: *B. cereus* CHU4R primarily targets the C-H bonds in CH_2_ groups, while *P. otitidis* YUC44 and *A. baumannii* YUCAN predominantly attack the ester bonds in the PBS structure.

#### 3.3.3. Analysis by ASAP-MS

**Figure 8:**
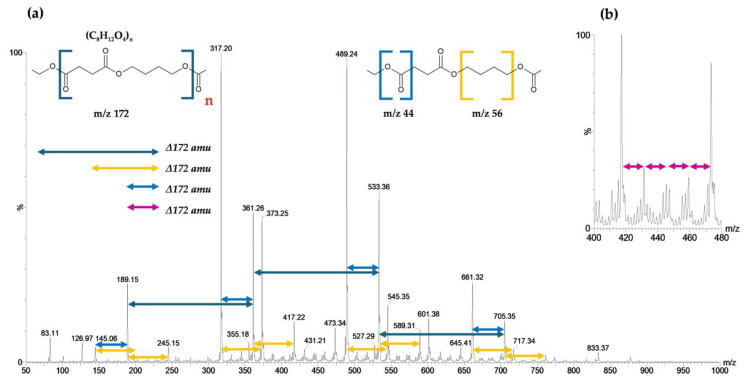
(**a**) ASAP-MS^(-)^ spectrum of PBS showing three ion distributions: the main series (indicated with arrows) with a mass difference of 172.1 amu, the secondary series of PBS–COO^−^ with a mass difference of 44 amu, and a third series of PBS + C_4_H_8_ (indicated with arrows) with a mass difference of 56 amu. (**b**) Section of the ASAP-MS spectrum highlighting the ion distribution with a mass difference of 14 amu.

The mass spectra of PBS obtained via ASAP-MS (Figure 8) revealed three ion series with a mass difference of 172.1 amu. The main series began at *m*/*z* 189.15, followed by a series corresponding to PBS–COO^−^ (with a 44 amu difference), starting at *m*/*z* 145.06. Additionally, two series corresponding to PBS + C_4_H_8_ (56 amu) were observed, with peaks at *m*/*z* 245.15 and *m*/*z* 201.06, the latter exhibiting lower intensity. These findings align with those reported by Barrère [31], who utilized ASAP-IM-MS for the characterization of biodegradable polymers such as PLA, PBS, and PE, suggesting that the observed mass differences could be attributed to the presence of copolymers in the material.

**Figure 9:**
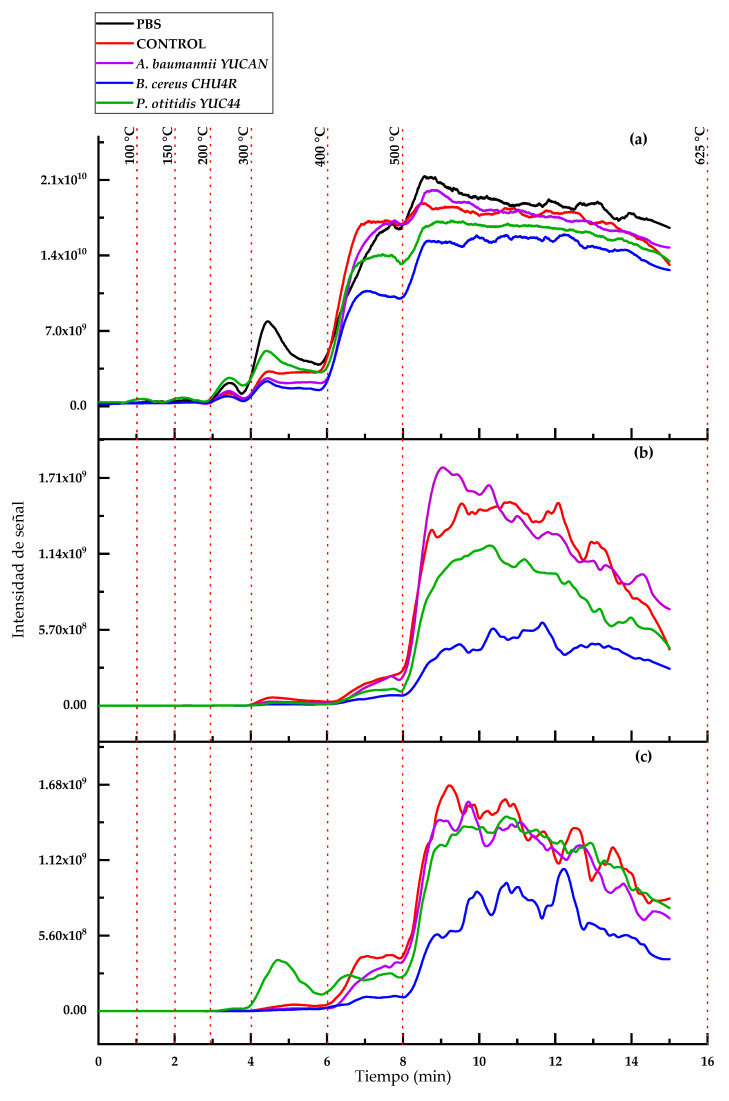
Chronograms and ASAP-MS temperature profile and of PBS films after 21 weeks of treatment: (**a**) total ions, (**b**) ion *m*/*z* 317, and (**c**) ion *m*/*z* 489.

On the other hand, the thermal profile and ion chromatogram (Figure 9) displayed a general decrease in intensity across different temperatures throughout the thermal ramp. The degradation order of the treated films was as follows: *B. cereus* CHU4R < *P. otitidis* YUC44 < PBS < A. *baumannii* YUCAN < control (Figure 9a). Additionally, the ion chromatograms and thermal profiles at *m*/*z* 317 and 489 (Figure 9b,c) exhibited a significant reduction in intensity between minutes 8 and 10 (Figures S2–S5). This phenomenon suggests that thermal degradation intensifies within this interval, which can be attributed to the degradation of the lower-molecular-weight fractions of PBS, therefore indicating that the microorganisms likely degraded lower-molecular-weight PBS molecules. Notably, the films treated with *B. cereus* CHU4R showed the greatest reduction in signal intensity.

**Figure 10:**
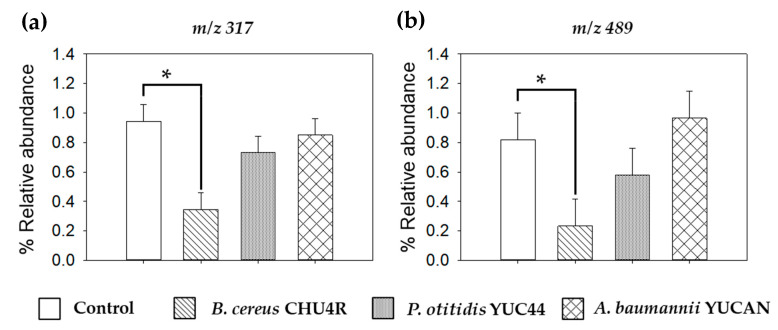
Relative abundance of the signal detected at (**a**) *m*/*z* 317 and (**b**) *m*/*z* 489 from the ASAP-MS spectra obtained from minutes 8 to 10 of the films after 21 weeks of treatment. * *p* < 0.05.

A detailed analysis of the ASAP-MS spectra within this period confirmed a significant decrease in the relative abundance of ions detected at *m*/*z* 317 and 489 in films treated with *B. cereus* CHU4R. In contrast, films treated with *A. baumannii* YUCAN and *P. otitidis* YUC44 showed no significant differences compared with the control (Figure 10). This could suggest that *B. cereus* CHU4R and *P. otitidis* YUC44 exert a more intense degradative effect, likely through specific enzymatic mechanisms that facilitate the cleavage of the PBS chain, which aligns with the observations in SEM (Figure 5), FTIR (Figure 7), as well as the observed changes in PBS films after 21 weeks of degradation (Figure 4). The absence of significant changes under other conditions supports the hypothesis that these microorganisms may degrade PBS through different pathways or with lower efficiency.

Finally, the comparative analysis reaffirms the capability of the ASAP-MS method to detect and differentiate structural modifications in biodegradable polymers such as PBS. The identification of specific ionic patterns (172.1, 44, 56, and 14 Da), in conjunction with the characterization techniques used in this study (FTIR, SEM, and degradation assays, among others), provides insights into the possible fragmentation and degradation mechanisms. These findings suggest that the loss of functional groups and the incorporation of additional units could be attributed not only to thermally induced ionization but also to enzymatic processes involved in biodegradation by different microorganisms. However, future studies should focus on evaluating the direct correlation between microbial enzymatic activity and the fragmentation patterns observed in ASAP-MS.

These findings have significant implications for the development and quality control of biodegradable materials. The ability to monitor degradation at the molecular level using techniques such as ASAP-MS enables a more precise evaluation of the degradative capabilities of different microorganisms and facilitates the identification of molecules generated during their degradation processes.

## 4. Conclusions

This study sought to assess the degradation capacity of PBS films, without any pre-treatment, by microbial consortia found in plastic waste in Yucatán. Five new strains with degradative activity were identified through BLAST analysis, and the sequences were registered in the NCBI repository.

The use of multiple characterization techniques, including ASAP-MS, SEM, FTIR, and degradation assays, allowed for a comprehensive evaluation of the PBS degradation activity of three of the five isolated strains (*A. baumannii* YUCAN, *B. cereus* CHU4R, *P. otitidis* YUC44) from different perspectives while maintaining consistency among the methods. The three strains showed the capacity and affinity to degrade different fractions of the PBS structure, suggesting that further degradation strategies could be explored in stages or by co-culture or microbial consortia.

To the best of our knowledge, this study represents the first report of plastic degradation activity in *A. baumannii* YUCAN, *P. otitidis* YUC44, and *B. cereus* CHU4R isolated in Yucatán. This research marks an important step toward developing sustainable strategies for plastic degradation and subsequent recovery. However, further studies are needed to optimize culture conditions and enhance the efficiency of the degradation process.

## 5. Patents

A patent application for this work, titled “*MICROORGANISMOS DEGRADADORES DE MATERIALES PLÁSTICOS*” was filed with the Mexican Institute of Industrial Property (Instituto Mexicano de la Propiedad Industrial, IMPI) on 14 December 2023, under application number 162899.

## Figures and Tables

**Table 1:** Tentative identification of isolated strains through 16S rDNA sequence analysis using BLAST.

Isolated Strain	Similarity (%)	Tentative Identity	NCBI Accession Number Assigned
MORI88	100	*Bacillus* sp.	NA
SIE11	100	*Bacillus* sp.	NA
CHU22	100	*Bacillus* sp.	NA
CHU4R	99.69	*Bacillus cereus*	ON721265
SIE4AN	100	*Bacillus* sp.	NA
MORI66	99.31	*Enterobacter hormaechei*	ON721279
SIE33	100	*Acinetobacter* sp.	NA
SIE22	99.71	*Acinetobacter* sp.	NA
MORI77	100	*Acinetobacter baumannii*	NA
YUCAN	100	*Acinetobacter baumannii*	ON721281
YUC99	100	*Klebsiella* sp.	NA
MORI33	100	*Klebsiella pneumoniae*	ON721265
MORI22	100	*Pseudomonas* sp.	NA
YUC44	99.90	*Pseudomonas otitidis*	ON721280

NA: not applicable.

## Data Availability

The original data presented in the study are openly available in the NCBI repository at: https://www.ncbi.nlm.nih.gov/nuccore/ON721265 (accessed on 27 September 2023); https://www.ncbi.nlm.nih.gov/nuccore/ON721279 (accessed on 7 July 2022); https://www.ncbi.nlm.nih.gov/nuccore/ON721281 (accessed on 7 July 2022); https://www.ncbi.nlm.nih.gov/nuccore/ON721265 (accessed on 7 July 2022); https://www.ncbi.nlm.nih.gov/nuccore/ON721280 (accessed on 7 July 2022).

## Supplementary Materials

The following supporting information can be downloaded at: https://www.mdpi.com/article/10.3390/polym17081128/s1. Table S1: Biochemical characterization for Gram staining, determination of spore production, catalase enzyme production, and methyl blue staining of the isolated strains; Figure S1: Location of sampling areas for the collection of microbial consortia: (a) Sierra Papacal Road, (b) Chuburná Puerto, (c) Yucalpetén; Figure S2: (a) ASAP-MS spectrum obtained from minutes 8 to 10 of PBS films treated with control after 21 weeks of treatment; Figure S3: (a) ASAP-MS spectrum obtained from minutes 8 to 10 of PBS films treated with *B. cereus* CHU4R after 21 weeks; Figure S4: (a) ASAP-MS spectrum obtained from minutes 8 to 10 of PBS films treated with *P. otitidis* YUC44 after 21 weeks; Figure S5: (a) ASAP-MS spectrum obtained from minutes 8 to 10 of PBS films treated with *A. baumannii* YUCAN after 21 weeks. References [60,61,62,63,64,65,66] are cited in the Supplementary Materials.
