# Supplementary material for: Isolation, Identification and Screening of Plastic-Degrading Microorganisms: Qualitative and Structural Effects on Poly(Butylene Succinate) (PBS) Films
Source: Polymers (Basel). 2025 Apr 21;17(8):1128. doi: 10.3390/polym17081128 (PMC12031546; doi:10.3390/polym17081128)
Supplement: Supplementary file 1 [file polymers-17-01128-s001.zip › polymers-3527827-supplementary.pdf]

Article

Isolation, Identification and Screening of Plastic-Degrading Microorganisms: Qualitative and Structural Effects on Poly(Butylene Succinate) (PBS) Films

Cristina América Morando-Grijalva [1], Ana Ramos-Díaz [1], Angel H. Cabrera-Ramirez [1], Juan Carlos Cuevas-Bernardino [2], Soledad Cecilia Pech-Cohuo [3], Angela Francisca Kú-González [4], Julia Cano-Sosa [1], Iván Emanuel Herrera-Pool [1], Sergio Valdivia-Rivera [2], Teresa Ayora-Talavera [1] and Neith Pacheco [1,*]

[1] Centro de Investigación y Asistencia en Tecnología y Diseño del Estado de Jalisco, A.C. Parque Científico Tecnológico de Yucatán, Km 5.5 Carretera, Sierra Papacal-Chuburna, Chuburna, Merida 97302, Yucatan, Mexico

[2] SECIHTI-Centro de Investigación y Asistencia en Tecnología y Diseño del Estado de Jalisco, A.C. Parque Científico Tecnológico de Yucatán, Km 5.5 Carretera, Sierra Papacal-Chuburna, Chuburna, Merida 97302, Yucatan, Mexico

[3] Departamento de Ingeniería en Robótica Computacional, Universidad Politécnica de Yucatán, Tablaje Catastral 7193, Carretera, Merida-Tetiz Km.4.5, Merida 97357, Yucatan, Mexico

[4] Unidad de Biología Integrativa, Centro de Investigación Científica de Yucatan, Merida 97205, Yucatan, Mexico

* Correspondence: npacheco@ciatej.mx; Tel.: +52-(33)-33455200 (ext. 4024)

Table S1: Biochemical characterization for Gram staining, determination of spore production, catalase enzyme production, and methyl blue staining of the isolated strains.

**Isolated strains**	**Cellular morphology**	**Gram staining**	**Spore production**	**Catalase**	**Reference**
Bacillus sp. MORI88	Bacillus	+	+	+	[1]
Bacillus sp. SIE11	Bacillus	+	+	+	[1]
Bacillus sp. CHU22	Bacillus	+	+	+	[1]
Bacillus cereus CHU4R	Bacillus	UC	+	+	[1]
Bacillus sp. SIE4AN	Bacillus	+	+	+	[1]
Enterobacter hormaechei MORI66	Bacillus	-	N.S.P.	+	[2]
Acinetobacter sp. SIE33	Bacillus	-	N.S.P.	+	[3]
Acinetobacter sp. SIE22	Bacillus	-	N.S.P.	+	[3]
Acinetobacter baumannii MORI77	Bacillus	-	N.S.P.	+	[4]
Acinetobacter baumannii YUCAN	Bacillus	-	N.S.P.	+	[4]
Klebsiella sp. YUC99	Bacillus	-	N.S.P.	+	[5]
Klebsiella pneumoniae MORI33	Coccobacillus	-	N.S.P.	+	[3]
Pseudomonas sp. MORI22	Bacillus	-	N.S.P.	+	[6]
Pseudomonas otitidis YUC44	Bacillus	-	N.S.P.	+	[7]

+: positive result; -: negative result; UC: unstained cells; N.S.P.: non-spore producing strain.

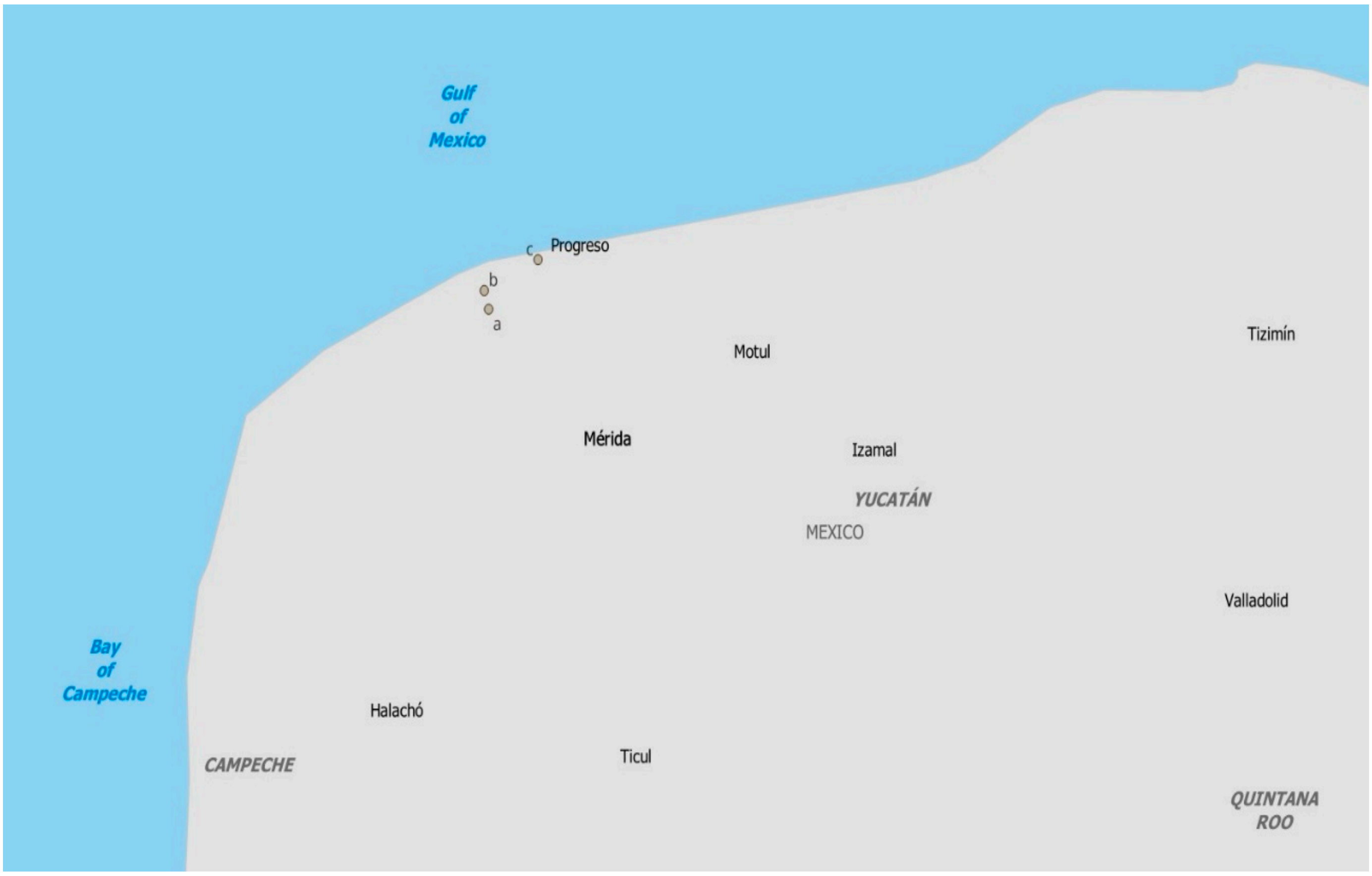

Figure S1: Location of sampling areas for the collection of microbial consortia: a) Sierra Papacal Road, b) Chuburná Puerto, c) Yucalpetén.

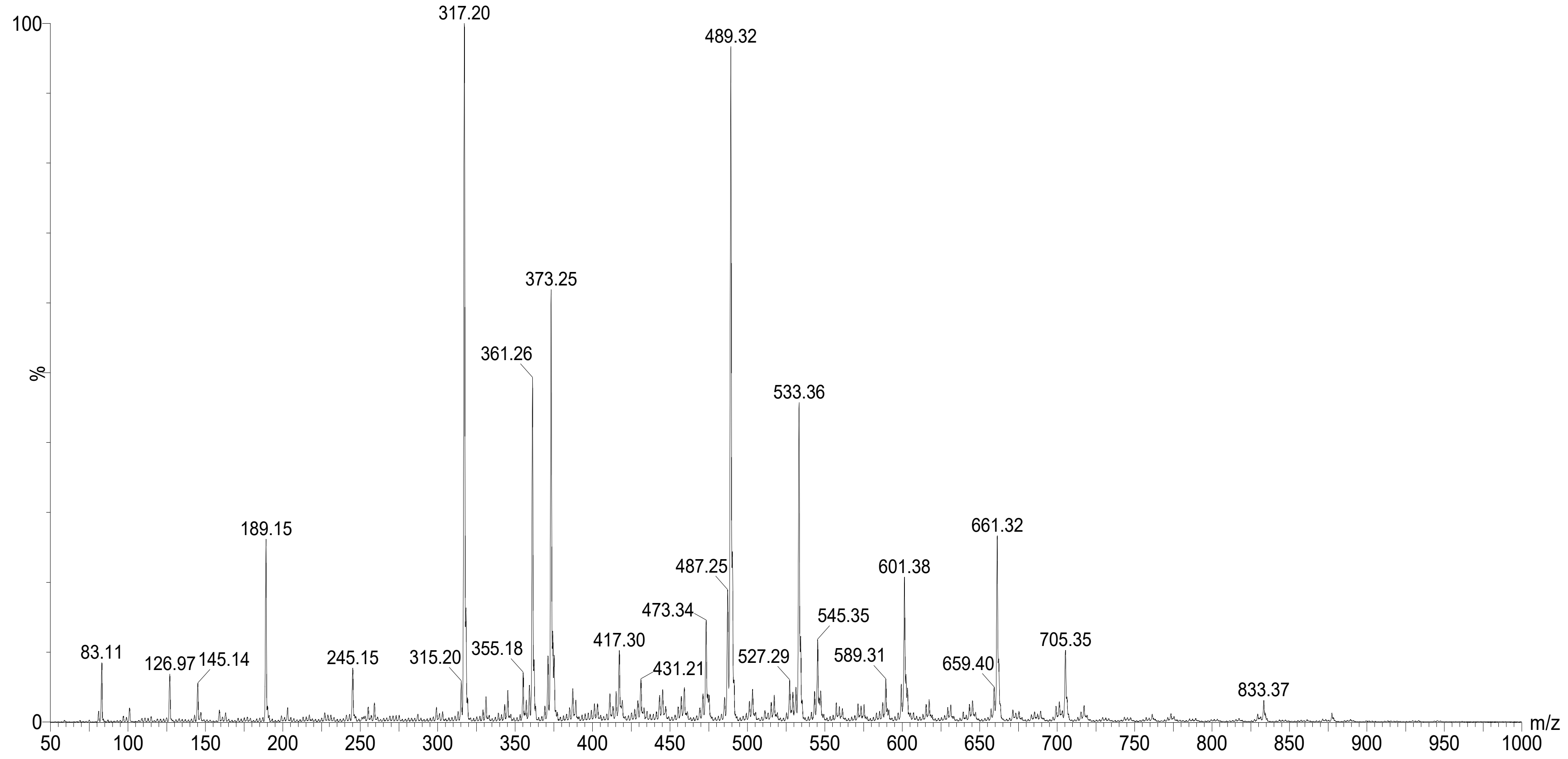

Figure S2: a) ASAP-MS spectrum obtained from minute 8 to 10 of PBS films treated with control after 21 weeks of treatment.

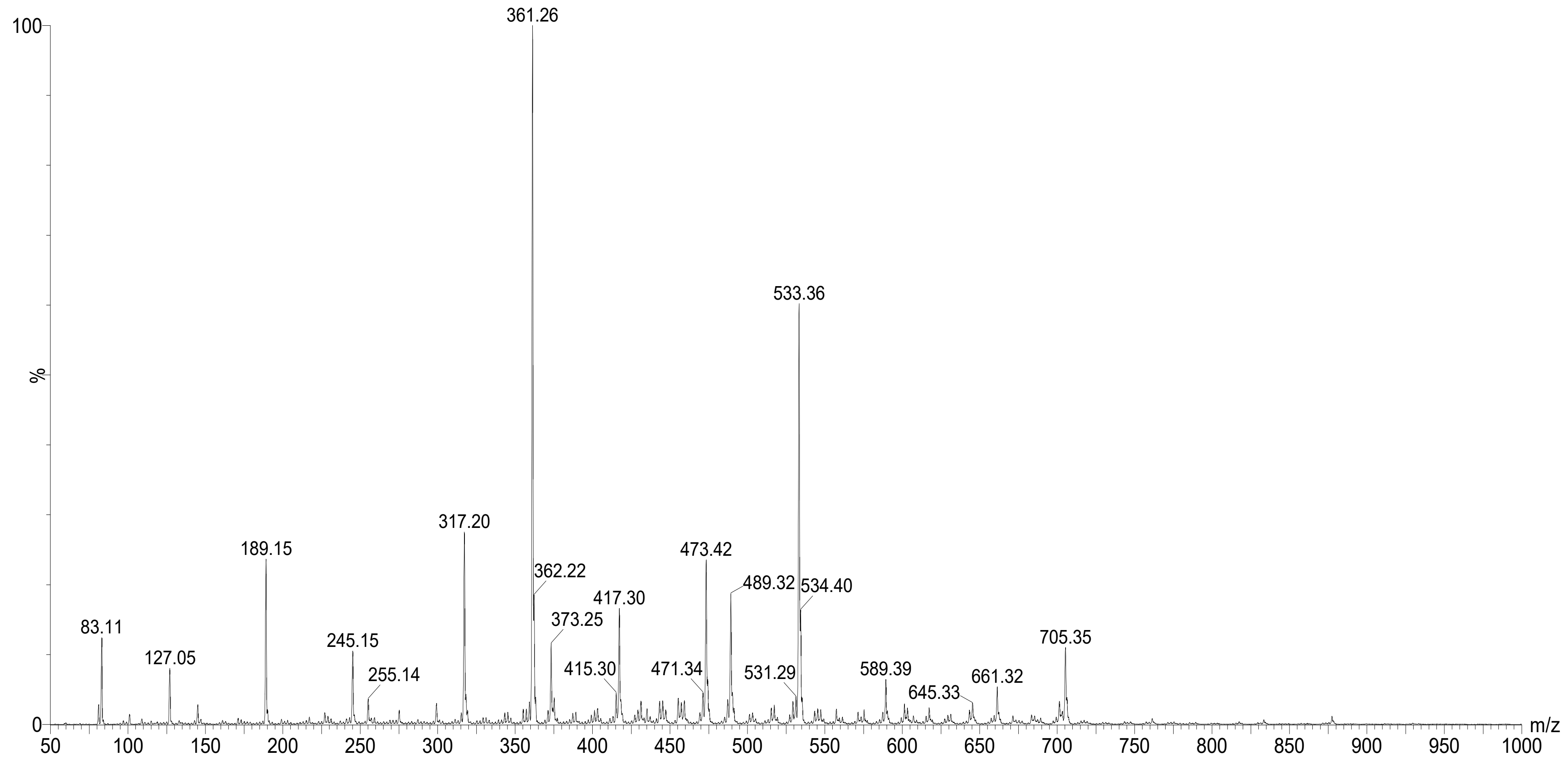

Figure S3: a) ASAP-MS spectrum obtained from minute 8 to 10 of PBS films treated with *B. cereus* CHU4R after 21 weeks.

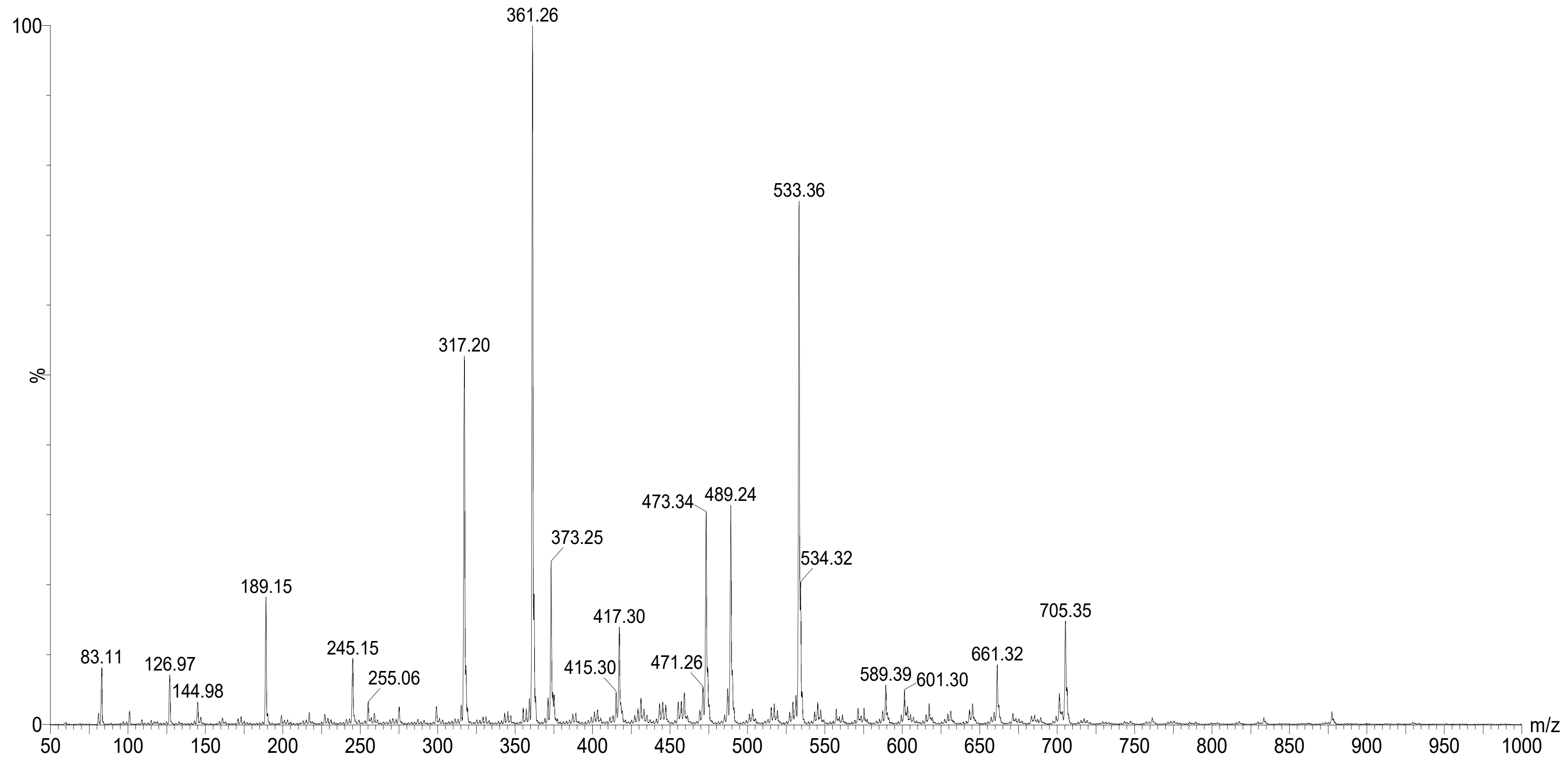

Figure S4: a) ASAP-MS spectrum obtained from minute 8 to 10 of PBS films treated with *P. otitidis* YUC44 after 21 weeks.

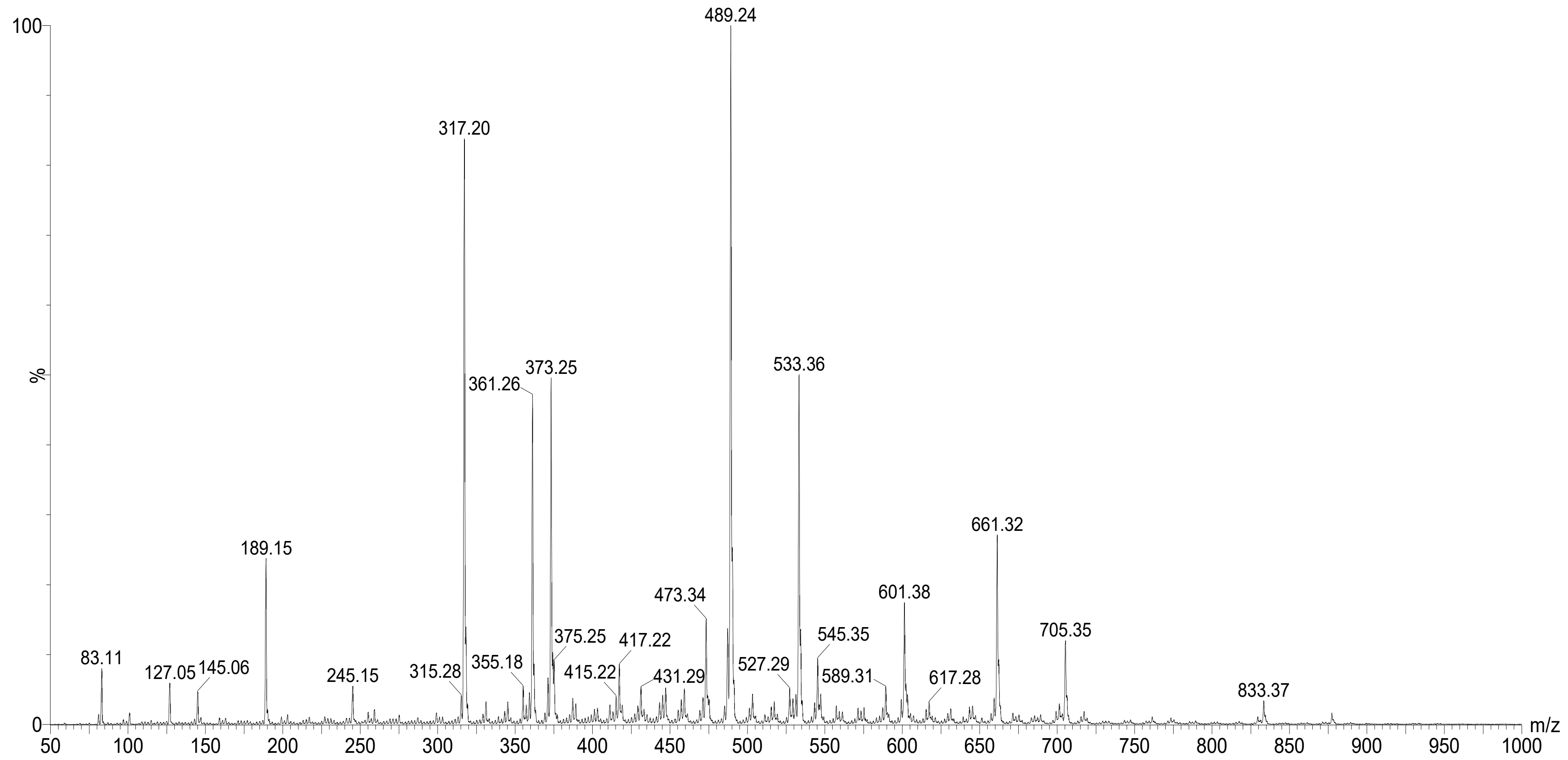

Figure S5: a) ASAP-MS spectrum obtained from minute 8 to 10 of PBS films treated with *A. baumannii* YUCAN after 21 weeks.

References

1. Gupta, R.S.; Patel, S.; Saini, N.; Chen, S. Robust demarcation of 17 distinct bacillus species clades, proposed as novel bacillaceae genera, by phylogenomics and comparative genomic analyses: Description of robertmurraya kyonggiensis sp. nov. and proposal for an emended genus bacillus limiting it o. *Int. J. Syst. Evol. Microbiol.* **2020**, *70*, 5753–5798, doi:10.1099/ijsem.0.004475.
2. Hoffmann, H.; Stindl, S.; Ludwig, W.; Stumpf, A.; Mehlen, A.; Monget, D.; Pierard, D.; Ziesing, S.; Heesemann, J.; Roggenkamp, A.; et al. Enterobacter hormaechei subsp. oharae subsp. nov., E. hormaechei subsp. hormaechei comb. nov., and E. hormaechei subsp. steigerwaltii subsp. nov., three new subspecies of clinical importance. *J. Clin. Microbiol.* **2005**, *43*, 3297–3303, doi:10.1128/JCM.43.7.3297-3303.2005.
3. Schoch, C.L.; Ciufo, S.; Domrachev, M.; Hotton, C.L.; Kannan, S.; Khovanskaya, R.; Leipe, D.; McVeigh, R.; O'Neill, K.; Robbertse, B.; et al. NCBI Taxonomy: A comprehensive update on curation, resources and tools. *Database* **2020**, *2020*, 1–21, doi:10.1093/database/baaa062.
4. Bouvet, P.J.M.; Grimont, P. a D. Taxonomy of the Genus Acinetobacter with the Recognition of nov . and Emended Descriptions of Acinetobacter calcoaceticus and Acinetobacter lwofii. *Int. J. Syst. Bacteriol.* **1986**, *36*, 228–240, doi:10.1099/00207713-36-2-228.
5. Rosenblueth, M.; Martínez, L.; Silva, J.; Martínez-Romero, E. Klebsiella variicola, A Novel Species with Clinical and Plant-Associated Isolates. *Syst. Appl. Microbiol.* **2004**, *27*, 27–35, doi:10.1078/0723-2020-00261.
6. Yang, G.; Han, L.; Wen, J.; Zhou, S. Pseudomonas guangdongensis sp. nov., isolated from an electroactive biofilm, and emended description of the genus Pseudomonas Migula 1894. *Int. J. Syst. Evol. Microbiol.* **2013**, *63*, 4599–4605, doi:10.1099/ijs.0.054676-0.
7. Clark, L.L.; Dajcs, J.J.; McLean, C.H.; Bartell, J.G.; Stroman, D.W. Pseudomonas otitidis sp. nov., isolated from patients with otic infections. *Int. J. Syst. Evol. Microbiol.* **2006**, *56*, 709–714, doi:10.1099/ijs.0.63753-0.
